# Socio-demographic disparities in receipt of clinical health care services during the COVID-19 pandemic for Canadian children with disability

**DOI:** 10.1186/s12913-022-08672-1

**Published:** 2022-11-28

**Authors:** Miriam Gonzalez, Jinan Zeidan, Jonathan Lai, Afiqah Yusuf, Nicola Wright, Mandy Steiman, Arun Karpur, Andy Shih, Mayada Elsabbagh, Keiko Shikako

**Affiliations:** 1 Faculty of Medicine, Montreal Neurological Institute-Hospital, McGill University, Rue University, 3775, H3A 2B4 Montréal, Canada; 2 Research Institute of the McGill University Health Centre, 1001 Decarie Blvd, H4A 3J1 Montréal, Canada; 3 Autism Alliance of Canada, 1111-23 Sheppard Ave E, M2N OC8 Toronto, ON Canada; 4 Department of Biostatistics and Health Informatics, King’s College London, WC2R 2LS London, UK; 5 Autism Speaks, 1060 State Rd #1446, 08540 Princeton, Princeton, NJ, NJ USA; 6 Autism Speaks, New York 1 E 33rd St, 10016 New York, NY USA; 7 School of Physical and Occupational Therapy, Faculty of Medicine, McGill University, Montréal, Canada

**Keywords:** COVID-19, Receipt of health services, Disparities, Neurodevelopmental conditions, Canadian families

## Abstract

**Background:**

Little is known about the experience of receiving in-person and virtual clinical health care services during the COVID-19 pandemic for Canadian children with developmental disabilities and delays facing multiple layers of vulnerability (e.g., low income, low educational attainment families). We examined the relationship between socio-demographic factors and the receipt of these services (physical and mental health services) during COVID-19 for Canadian children with these conditions.

**Methods:**

Data collected in Canada for the Global Report on Developmental Delays, Disorders and Disabilities were used. The survey: (1) was developed and disseminated in collaboration with caregivers of children with disabilities, (2) included topics such as response to the pandemic and receipt of services and supports, and (3) documented the experiences of a non-random convenience sample of caregivers of children (any age) with these conditions during and prior to the pandemic. We used four logistic regression models to assess the association between socio-demographic factors and receipt of services.

**Results:**

Being a single parent, having low educational attainment (high school or less), having low income (making less than $40,000 per year), working less than full time (working part-time, working reduced hours due to COVID, retired, stay home parent or student), as well as male gender and older age of the child with disability were factors associated with decreased likelihood of receiving services.

**Conclusion:**

Our findings point to the need for tailoring services for families of children with disabilities, particularly low socioeconomic status families, to ensure continuity of care during public health emergencies.

## Background

The COVID-19 pandemic and related preventative measures put in place to save lives have posed considerable challenges to a vulnerable group of Canadians: children with developmental disabilities and delays (DDDs)[1–3]. DDDs are a group of disorders characterized by early onset, high incidence of comorbidity, and a variety of activity limitations in areas such as learning, social behaviour, and motor development [4]. Children with DDDs may be at increased risk of exposure, contraction, and susceptibility to COVID-19 given their underlying health conditions, living arrangements in some cases (e.g., children in group homes or care facilities), and their dependency on support workers and health and community-based services [5]. Strategies used to contain the spread of the Sars-Cov2 virus (e.g., lockdown, reducing number of physical contacts) have potential side effects (e.g., social isolation, mental health distress) that may harm this population to a greater degree. Despite these potential negative impacts, increased vulnerability, and a greater need for a wide range of services, not all these children are receiving clinical health care services during the pandemic. For instance, a report based on crowdsourced data on the impact of the pandemic on caregivers of children with DDDs from across Canadian provinces and territories revealed that almost half of those who completed the survey reported not receiving services in a clinic for their child’s physical health (44.4%) or their child’s mental health (46.1%) [6]. About a third also reported not having received telehealth services for their child’s physical health (29.5%) or mental health (33.4%). Yet, many caregivers reported changes in their child’s functioning: worsening sleep problems, mental health problems, and repetitive behaviours were the top three conditions that had worsened [6].

Lack of service use during the pandemic and resulting omission or delay of appointments can have short and long-term detrimental consequences on the health and wellbeing of children with DDDs. Yet the needs of those with disability are often inadequately addressed during public health emergencies [7]. Indeed, the growing body of literature suggests the COVID-19 pandemic, like previous pandemics, has exacerbated pre-existing inequities [8–11], placing unequal health, social, and economic burden on socially disadvantaged groups such as those with disability, racial and ethnic minorities, and low-income persons [1, 10–15].

Various frameworks propose ways of understanding (in)equities and resulting disparities among groups of people [16–19]. Saint Girons and authors [9] propose a model for understanding drivers of (in)equity during a pandemic that outlines how structural drivers (e.g., structural racism), life conditions (e.g., income), governance (e.g., governance systems), and observance of human rights (e.g., standards and commitment) influence health (in)equity [9]. The model also recognizes the contributing role of gender, sexuality, ethnicity, disability, migration, and their intersectionality. Inequities specific to the COVID-19 pandemic outlined in the model include: impact of containment measures (e.g., pre-existing socioeconomic conditions), exposure to the virus (e.g., occupation type), susceptibility to the virus (e.g., pre-existing health conditions), and access to treatment (e.g., availability of services).

Although there is a dearth of research on factors that influence receipt of services for children with DDDs during pandemics, the emerging literature on disparities in service access, quality, and utilization for children with DDDs pre-pandemic suggests that receiving services during public health emergencies may be influenced by sociodemographic factors. For instance, in high-income countries being non-white has been associated with: (1) decreased health services use and access [20], reduced likelihood of receiving family-centred care [21–25] or using subspecialty care [26], (2) greater odds of having problems accessing healthcare [20, 25, 27, 28], (3) receiving a diagnosis at an older age [29, 30], and (4) receiving poorer quality of care [20, 21, 24, 31, 32].

Low socioeconomic status, characterized as low family income or low educational attainment, has similarly been associated with decreased access to health services [22, 25, 33–35], decreased health services utilization [34, 36], reduced likelihood of receiving family centred care [24, 25, 37] or using specialty services [24, 38], receiving a diagnosis at an older age [39–41], and receiving poor quality care [24, 33].

The objective of the present study was to examine how socio-demographic factors are related to receipt of clinical health care services (physical and mental health services) during the COVID-19 pandemic for Canadian children with developmental disabilities and delays. Examining the distinct experiences of families affected by disability in the Canadian context is crucial for developing effective public health responses, policies, and needed supports during the pandemic and beyond. We used the model proposed by Saint Girons and authors [9] as a guiding framework as this model highlights drivers of inequities in the context of pandemics.

## Methods

### Data

We used data collected in Canada for the Global Report on Developmental Delays, Disorders and Disabilities. The Global Report is an ongoing initiative led by the World Health Organization (WHO), UNICEF, and Autism Speaks to document the experiences of caregivers of children with these conditions around the world. In Canada, the Global Report Survey was designed to document the experiences of caregivers of children (any age) with these conditions during and prior to the COVID-19 pandemic. Survey questions were based on COVID-19 policy guidance recommendations for persons with disabilities [42–45] and included topics such as response to the pandemic and receipt of services and supports. The survey was developed, tested, and disseminated in collaboration with caregivers of children with DDDs and was available in both English and French. An informed consent statement preceded the survey. Ethics approval was obtained from the McGill University’s Research Ethics Board.

The Global Report Survey used a cross-sectional design and an online convenience sampling strategy. Various dissemination strategies were used: (1) researchers, organizations, research centres, and research networks across Canadian provinces and territories were sent information about the study as well as the recruitment flyer and were invited to disseminate the survey via their networks (e.g., email distribution lists, newsletters, research websites), (2) patient-partners shared the survey through their network of caregivers, and (3) team members shared the link to the survey through social media platforms such as Facebook and Twitter. The survey was active from June to July 2020 and collected information from a non-random, convenience sample of caregivers of children (of any age) with these conditions from across Canadian provinces and territories. A total of 2,133 responses were received and verified for invalid responses resulting in a total of 883 valid responses [for information about the data validation process, see Gonzalez and authors [6]]. Caregivers were offered $15 in appreciation for their participation.

### Variables selected for the Present Study

**Outcome variables**. To measure receipt of clinical health care services for the child with disability, the following four items were used: “During the pandemic, have you gotten enough information, services, or support in the following areas for your child?”: (1) “Services for your child’s physical health in a clinic”, (2) “Services for your child’s mental health in a clinic”, (3) “Services for your child’s physical health with a professional over the phone or video calls”, and (4) “Services for your child’s mental health with a professional over the phone or video”.

Respondents could select: “I got none”, “I got some but not enough”, and “Enough”. The item: “I got none” was recoded to a value of 0 indicating “No, services had not been received”. The remaining items were recoded to a value of 1 indicating “Yes, services had been received”.

**Predictor variables**. Child age and gender, caregiver ethnicity, education, work status, and province of residence, household type and income were selected for the multivariate analysis. Child age measured age in years. Other variables were recoded based on the data distribution: Child gender was recoded such that a value of 1 corresponded to male and a value of 0 corresponded to female. Caregiver ethnicity was recoded into three categories: Caucasian, Indigenous, and other ethnicity. The education variable was recoded into the following categories: High school or less, diploma or undergraduate degree, and graduate education or professional. The work status variable included the following categories: working full time, working part-time, stopped working or reduced hours due to the COVID-19 pandemic, and retired/stay at home parent/student. Caregiver province of residence was recoded to include the following categories: British Columbia, Alberta, Prairies (Manitoba and Saskatchewan), Ontario, Quebec, the Territories (Northwest Territories, Nunavut, and Yukon), and the Atlantic provinces (Nova Scotia, New Brunswick, Prince Edward Island, Newfoundland and Labrador). The Household type variable was recoded to include the following categories: single parent household and two-parent household. Finally, the income variable was recoded into three categories: less than $40,000, between $40,000 and $80,000, and more than $80,000 per year. In the multivariate analyses, the following were used as the reference categories: male, Caucasian, Quebec, less than high school, working full-time, two-parent household, and an income greater than $80,000.

### Data analyses

We used Stata/MP 13.1. to conduct the data analysis [46]. The data were first examined to verify that all assumptions for logistic regression analysis (e.g., multicollinearity among independent variables, linearity of independent variables and log odds) were met [47]. Sample characteristics were then explored through univariate analysis and the relationships between predictor and outcome variables were examined using chi-square tests of significance. Four separate binary logistic regressions were used to explore the associations between socio-demographic characteristics and receipt of services (in-clinic and telehealth services). Predictor variables were entered using a simultaneous approach while controlling for child age and gender. Cases with missing data were excluded from the analysis.

## Results

The final sample consisted of 797 caregivers (see Table 1). Approximately 96% of the children in the sample were 21 years of age or younger (mean age: 9 years). Over half of the caregivers were Caucasian (61.86%), had a diploma or undergraduate degree (65.50%), worked full time (50.19%), and reported making between $40,000-$79,999 a year (52.70%). Almost half reported not having received in-clinic services for their child’s physical health (45.92%) and mental health (47.30%).

**Table 1:** Descriptives for predictor and outcome variables (N = 797)

Variable	Mean (range) or %
**Child age**	9 (0-41yrs)
0-5years	22.84
6-12years	56.97
13–21 years	16.18
≥ 22 years	4.04
**Child gender**	
Female	42.41
Male	57.59
**Caregiver ethnicity**	
Caucasian	61.86
Indigenous	24.72
Other (Asian, Arab, Latin-American, Black, Other)	13.43
**Caregiver education**	
High school or less	9.16
Diploma/Undergraduate	65.50
Graduate/Professional	25.35
**Caregiver work status**	
Working full time	50.19
Working part-time	17.06
Stopped working or Reduced hours due to COVID	19.45
Retired/Stay at home parent/Student	13.3
**Household type**	
Single parent household	18.95
Two parent household	81.05
**Household income**	
Less than $40,000	12.17
$40,000- $79,999	52.70
$80,000+	35.13
**Caregiver province of residence**	
Ontario	32.75
British Columbia	19.70
Quebec	19.57
Alberta	14.05
Manitoba/Saskatchewan	8.16
Newfoundland/Nova Scotia/Prince Edward Island	4.14
Northwest Territories/Nunavut/Yukon	1.63
**Receipt of in-clinic services for child physical health**	
Yes	54.08
No	45.92
**Receipt of online/telehealth services for child physical health**	
Yes	70.14
No	29.86
**Receipt of in-clinic services for child mental health**	
Yes	52.7
No	47.3
**Receipt of online/telehealth services for child mental health**	
Yes	65.87
No	34.13

Note. Data Source: data collected in Canada for the Global Report on Developmental Delays, Disorders and Disabilities, an initiative led by the World Health Organization, UNICEF, and Autism Speaks. The Global Report Survey in Canada used a cross-sectional design and an online convenience sampling strategy to document the experiences of caregivers of children (any age) with these conditions during and prior to the COVID-19 pandemic

Table 2 provides the results of the four logistic regressions on receipt of clinical health care services during the pandemic (in-clinic and telehealth services) for the child’s physical and mental health. Adjusted odds ratios, betas, and confidence intervals are reported. Child age was negatively associated (aOR = 0.97) with odds of receiving telehealth services for the child’s physical health. Having a child who was male decreased the odds of receiving telehealth services for the child’s physical health (aOR = 0.75) and in-clinic services for the child’s physical health (aOR = 0.74) and mental health (aOR = 0.74).

**Table 2:** Results of binary logistic regressions on receipt of services for the child (N = 797)

Variable		Receipt of Services											
		**Child Mental Health**						**Child Physical Health**					
		**In-clinic**			**Telehealth**			**In-clinic**			**Telehealth**		
		***B***	***aOR*** ^***1***^	***95% CI***	***B***	***aOR*** ^***1***^	***95% CI***	***B***	***aOR*** ^***1***^	***95% CI***	***B***	***aOR*** ^***1***^	***95% CI***
**Child age**		-0.02	0.98	(-0.05–0.01)	-0.01	0.99	(-0.04–0.01)	-0.02	0.98	(-0.05–0.01)	-0.03	0.97**	(-0.06 - -0.01)
**Child gender** (Ref^2^: female)		-0.31	0.74*	(-0.61–0.003)	-0.08	0.92	(-0.39–0.23)	-0.30	0.74*	(-0.60–0.002)	-0.29	0.75*	(-0.62–0.04)
**Caregiver ethnicity** (Ref^2^: Caucasian)													
	Indigenous	0.90	2.45***	(0.50–1.30)	0.73	2.07***	(0.31–1.14)	0.50	1.65***	(0.12–0.88)	0.59	1.80***	(0.15–1.02)
	Other	-0.33	0.72	(-0.80–0.13)	-0.12	0.88	(-0.57–0.33)	-0.16	0.85	(-0.61–0.29)	-0.31	0.73	(-0.77–0.15)
**Caregiver education** (Ref^2^: high school or less)													
	Diploma/Undergraduate	0.67	1.95**	(0.09–1.25)	0.12	1.13	(-0.44–0.67)	-0.17	0.85	(-0.71–0.38)	0.40	1.49	(-0.17–0.97)
	Graduate/Professional	0.75	2.11**	(0.10–1.39)	0.20	1.22	(-0.43–0.83)	-0.22	0.80	(-0.84–0.39)	0.49	1.63	(-0.16–1.14)
**Caregiver work status** (Ref^2^: working full time)													
	Working part-time	-0.77	0.47***	(-1.20 - -0.33)	-0.18	0.84	(-0.62–0.27)	-0.66	0.52***	(-1.08 - -0.24)	-0.54	0.58**	(-0.99 - -0.10)
	Stopped/Reduced hours due to covid	-0.64	0.53***	(-1.07 - -0.22)	-0.67	0.51***	(-1.09 - -0.24)	-0.79	0.45***	(-1.20 - -0.38)	0.32	1.38	(-0.15–0.79)
	Retired, Stay at home parent, Student	-0.47	0.63*	(-0.99–0.05)	-0.40	0.67	(-0.91–0.11)	-0.88	0.42***	(-1.39 - -0.37)	0.13	1.14	(-0.42–0.67)
**Household type** (Ref^2^: single parent household)													
	Two-parent household	0.24	1.27	(-0.17–0.65)	0.29	1.34	(-0.11–0.69)	-0.42	0.66**	(-0.82 - -0.01)	0.55	1.73***	(0.14–0.96)
**Household income** (Ref^2^: <$40,000)													
	$40,000-$79,999	0.47	1.60*	(-0.07–1.01)	-0.22	0.81	(-0.75–0.32)	0.17	1.18	(-0.36–0.69)	-0.36	0.70	(-0.93–0.21)
	$80,000+	0.16	1.17	(-0.44–0.75)	-0.30	0.74	(-0.88–0.28)	-0.03	0.97	(-0.60–0.54)	-0.64	0.53**	(-1.26 - -0.02)
**Caregiver province of residence** (Ref^2^: Quebec)													
	Ontario	0.49	1.64**	(0.06–0.92)	0.41	1.51*	(-0.01–0.83)	0.42	1.52*	(-1.72–0.83)	0.34	1.41	(-0.09–0.78)
	Alberta	0.74	2.09***	(0.22–1.26)	0.44	1.559*	(-0.08–0.97)	0.57	1.772**	(0.06–1.09)	0.27	1.30	(-0.27–0.80)
	British Columbia	0.85	2.33***	(0.34–1.35)	0.90	2.465***	(0.38–1.43)	0.61	1.845**	(0.12–1.10)	0.41	1.51	(-0.11–0.94)
	Manitoba/Saskatchewan	0.06	1.06	(-0.58–0.69)	0.18	1.198	(-0.43–0.79)	-0.01	0.993	(-0.63–0.61)	0.74	2.10**	(0.07–1.41)
	NFL/NS/PEI^3^	1.06	2.88**	(0.20–1.92)	0.09	1.093	(-0.72–0.90)	1.27	3.564***	(0.37–2.17)	1.83	6.25***	(0.58–3.09)
	NWT/NT/Yukon^4^	1.02	2.76	(-0.24–2.27)	0.55	1.730	(-0.70–1.80)	0.26	1.292	(-0.92–1.43)	0.16	1.17	(-1.10–1.41)
Constant		-1.00	0.37**	(-1.90 - -0.11)	0.38	1.464	-0.50–1.26	0.85	2.349*	(-0.01–1.72)	0.55	1.73	(-0.37–1.46)

Note. Data Source: data collected in Canada for the Global Report on Developmental Delays, Disorders and Disabilities, an initiative led by the World Health Organization, UNICEF, and Autism Speaks. The Global Report Survey in Canada used a cross-sectional design and an online convenience sampling strategy to document the experiences of caregivers of children (any age) with these conditions during and prior to the COVID-19 pandemic

^1^aOR= Adjusted odds ratio. ^2^Ref=Reference Category. ^3^NFL/NS/PEI = Newfoundland, Nova Scotia, Prince Edward Island. ^4^NWT/NT/Yukon = Northwest Territories, Nunavut, Yukon

*p < .05 **p < .001

Caregiver Indigeneity was associated with greater odds of receiving in-clinic and telehealth services for both mental and physical health. Compared to caregivers who reported being Caucasian, Indigenous caregivers were twice as likely to receive in-clinic (aOR = 2.45) and telehealth services (aOR = 2.07) for their child’s mental health and twice as likely to receive in-clinic (aOR = 1.65) and telehealth services (aOR = 1.80) for their child’s physical health.

Higher caregiver educational attainment was associated with greater odds of receiving in-clinic services for the child’s mental health. For instance, compared to caregivers with less than high school education, caregivers who reported having a diploma or undergraduate degree (aOR = 1.95) and those who reported having a graduate or professional degree (aOR = 2.11) were twice as likely to receive in-clinic services for their child’s mental health. Having a full-time job was also beneficial for receiving services. Compared to caregivers who reported working full- time, those working part-time had approximately half the odds of receiving telehealth services for their child’s physical health (aOR = 0.58) and in-clinic services for their child’s physical health (aOR = 0.52) or mental health (aOR = 0.47). Similarly, those who stopped working or worked reduced hours due to the COVID-19 pandemic had half the odds of receiving telehealth services for their child’s mental health (aOR = 0.51) and in-clinic services for their child’s mental health (aOR = 0.53) or physical health (aOR = 0.45) compared to those working full time. Finally, those who were retired, at home parents, or students were 0.63 times less likely to receive in-clinic services for their child’s mental health and 0.42 times less likely to receive in-clinic services for their child’s physical health than caregivers who reported working full time.

Compared to those living in single-parent households, those living in two-parent households were twice as likely (aOR = 1.73) to receive telehealth services for their child’s physical health and 0.66 times less likely to receive in-clinic services for their child’s physical health. Making between $40,000-$79,999 a year was associated with receiving in-clinic services for the child’s mental health. For instance, compared to those making less than $40,000 per year, those who reported making between $40,000–79,999 were twice as likely to receive in- clinic services for their child’s mental health (aOR = 1.60). Making $80,000 or more decreased the odds of receiving telehealth services for the child’s physical health by half (aOR = 0.53). Finally, residents of Ontario, Alberta, British Columbia, and the Atlantic provinces were more likely to receive almost all types of services as compared to Quebec residents. For instance, residents of the Atlantic provinces were over three times more likely (aOR = 3.56) to receive in-clinic services for their child’s physical health and six times more likely (aOR = 6.25) to receive telehealth services for their child’s physical health.

## Discussion

Despite Canada’s universal health coverage model, our findings reveal socio-demographic disparities in receipt of clinical health care services during the COVID-19 pandemic among Canadian children with developmental disabilities and delays. Working less than full time, having low educational attainment (high school or less), being a single parent, having low income (making less than $40,000 per year), as well as male gender and older age of the child with disability were factors associated with decreased receipt of services. Our findings contribute to existing literature in this area by confirming the differential impact of the COVID-19 pandemic on already disadvantaged families [1, 10, 14] and point to important public health considerations.

### Socio-Economic factors

We examined the relationship between receipt of services and proxies of socioeconomic status: caregiver work status, caregiver education, household composition, and household income. A work arrangement other than full-time work (working part-time, working reduced hours due to COVID, stopped working dure to COVID, retired, stay home parent, or student) was associated with decreased odds of receiving in-clinic and telehealth services for the physical and mental health of the child with disability. Research has found that parents of children with disabilities often reduce their work schedules to prioritize their child’s care [48] and have lower levels of workforce participation [49–51]. Reduction in work hours, cessation of paid employment, and the high costs associated with disability-related services may signify loss of income for these families. Lower household income has been associated with less likelihood of receiving needed services [24, 38] and accessing specialized services [22, 38]. Thus, families with more financial resources are more likely to obtain care for their children yet children with disabilities are more likely to live in financially disadvantaged households [48]. Initiatives designed to support families experiencing employment changes and possible financial strain are justified.

In terms of education, high educational attainment (more than high school) was associated with greater odds of receiving in-clinic services for the mental health of the child with disability. This finding is consistent with past research that has found higher parental education to increase the likelihood of receiving services such as early intervention services [52], non-traditional alternative services such as vitamin therapy [35, 36], as well as specialty services [35]. Parents with higher education have been found to use more types of services than less educated parents [25, 34]. It may be that parents with higher education have the financial resources and access to tools (e.g., internet, social networks) that facilitate searching for information about available services and seeking out needed services.

Similarly, living in a two-parent household was associated with greater odds of receiving telehealth services for the physical health of the child with disability. Although there is a dearth of research exploring the relationship between household composition and receipt of services for children with DDDs during pandemics, our finding is consistent with past research that has found two-parent households to be associated with receipt of care coordination services [24]. It may be that the advantages of living in two-parent households (e.g., higher household income, spousal support, shared responsibility of caring for the child) enable or empower caregivers to seek out alternative services. Living in a two-parent household was also associated with decreased odds of receiving in-clinic services for the physical health of the child. It may be that caregivers from two-parent households in our study were aware of service closures or disruptions and sought and used alternative, online services.

Finally, when compared to caregivers making less than $40,000 per year, those who reported making between $40,000–79,999 were twice as likely to receive in-clinic mental health services for the child with disability. This finding is consistent with past studies that have found that compared to families with lower income, families with annual incomes above $50,000 have higher odds of using specialized services such as speech language therapy and ABA [35, 53]. Higher income parents or caregivers may be: (1) more aware of where services are offered, (2) more proactive in looking for services, and may be (3) more likely to afford costs related to these services. The finding that making $80,000 or more per year was associated with decreased odds of receiving telehealth services for the physical health of the child with disability was surprising. This finding diverges from the existing literature that links higher income with greater likelihood of using specialized services [35, 53] and may be the result of differences in measures used across studies to capture low- versus high- income families [22, 53].

### Child and caregiver factors

Male gender of the child with disability decreased the odds of receiving telehealth and in-clinic services for physical health as well as in-clinic mental health services. Although research on sex differences in health service use or receipt for children with disability is limited [57], a study that examined and compared the service utilization experiences of females and males with autism spectrum disorder (ASD) found that adolescent girls used a wider range of services compared to adolescent boys [57]. Past research has also found that compared to males with neurodevelopmental conditions, females with these conditions experience higher rates of physical and mental health problems [58, 59]. It may be that the males with disability in our sample (57.59%) experienced lower rates of health problems and accessed less services compared to the females in our sample. Future research on how sex influences service receipt and/or use for this subgroup of the population is needed.

We also found an inverse negative relationship between child age and service receipt for physical health. This finding may be driven by less access to services for youth and adults with disability [60–63]. Older individuals with disability may not be aware of where to access services once they leave child and school-based services [64]. Understanding how service utilization and needs change with age requires further systematic inquiry.

Contrary to our expectations, we did not identify disparities by caregiver Indigeneity. This finding was surprising given past research documenting health disparities and inequitable access to healthcare services for Indigenous people [54–56]. Although reasons for this association are unclear, one explanation might be sampling bias. It is possible that subgroups connected with Indigenous disability organizations who have relatively more access to services were more likely to complete the survey.

Finally, the finding that residents of Ontario, Alberta, British Columbia, and the Atlantic provinces were more likely to receive almost all types of services compared to Quebec residents is in line with the finding that Quebec has worse access to primary care compared to other Canadian provinces [65]. However, this finding must be interpreted with caution given the skewed distribution of our sample (underrepresentation from the Atlantic provinces, the prairies, and the territories). Thus, the relationship between caregiver province of residence and receipt of services necessitates further investigation.

### Implications

Taken together, our findings point to the need to tailor clinical health care services for low socioeconomic status families of children with developmental disabilities and delays (DDD) to ensure continuity of care during public health emergencies. Past research has found decreased access and use of health care services for low socioeconomic status families [33, 34] and that these inequities are further exacerbated during pandemics [11, 12, 14]. Decreased access and use of health services for these families may be due to lack of: (1) awareness of available services, (2) financial means to pay for out-of-pocket services, or (3) flexible work schedules that allow them to focus on caregiving responsibilities. To address potential lack of awareness about available services, public health can ensure information about service disruptions and available services adhere to accessibility guidelines (e.g., available in various languages, culturally appropriate) and are readily available (e.g., pamphlets given to families at first point of contact). In this study, more than half of caregivers who completed the survey reported receiving telehealth services for the child physical health (70.14%) and mental health (65.87%). This suggests that virtual modes of service delivery were feasible for our sample. Further examination of telehealth access and quality for the general population of families of children with a DDD is warranted.

Supporting these families through policies and programs designed to mitigate negative impacts of the pandemic is also needed. For instance, employment policies regarding work schedules and caregiving leaves must be flexible (e.g., different intervals and settings) to accommodate workers’ needs [66]. Benefits or income supports introduced in Canada by the federal government in response to COVID-19 such as the Canada Emergency Response Benefit (CERB) or the Canada Recovery Caregiving Benefit (CRCB) must be: (1) accessible to all irrespective of work status (employed vs. not employed) at time of application, (2) sufficient to help applicants out of financial strain/poverty, and (3) extended beyond the public health emergency as a way of enhancing quality of life and promoting financial stability [66].

To adequately meet the needs of low socioeconomic status families of children with disability, emergency preparedness planning must include plans for: (1) providing accessible services to meet the needs of children with DDDs from diverse backgrounds, (2) a pandemic communication strategy (public health information) that is accessible (e.g., multiple languages, culturally appropriate) to ensure reaching families from diverse backgrounds, and (3) involving representatives from these disadvantaged communities (e.g., caregivers) in the planning and development of innovative solutions to facilitate access and continuity of services during the pandemic and beyond.

Finally, a recovery plan that is inclusive of those living with disability and multiple levels of vulnerability (e.g., low-income, low educational attainment) is also needed. Meaningful engagement or collaboration with representatives from these communities in all stages of recovery planning, implementation, and before emergencies strike can contribute to an ongoing understanding of the needs of this subgroup and can shape healthcare programs and responses. For instance, engagement of representatives from these communities in surveillance planning or collection of disaggregated data (by disability, sex, age, etc.) on pandemic impact can facilitate a better assessment of families’ current realities and ongoing needs. The need to collect concrete indicators on the health and well-being of children with disabilities in Canada has been brought up by the UN Committee on the Convention of the Rights of Persons with Disabilities [67] and through the UNICEF reports that consistently show poor outcomes for this population [68]. The ability to respond adequately and provide necessary services that account for the multiple realities of vulnerable families across the country depends on careful planning and collaboration with these families.

## Limitations and future directions

One limitation of this study is that our findings are based on cross-sectional data. That is, findings represent a snapshot of caregivers’ experiences from June to July 2020 and do not allow for assessment of current or long-term pandemic impact on receipt of services. Second, our use of a convenience sample with inherent bias potential (e.g., sampling bias, selection bias) limits generalizability of findings to the general population of families of children with a DDD. Further, over half of survey respondents were white and indicated having a university degree. Thus, the sample was not representative of the Canadian parent population of children with DDDs. Third, only 4.04% of the children in our sample were 22 years of age or older. Examination of the experiences of families of young adults with DDDs is warranted. Fourth, interpretation of findings regarding province of residence should be made with caution due to the skewed distribution of our sample with greater representation from four provinces: Quebec, Ontario, Alberta, and British Columbia. Fifth, the survey was only available online limiting the participation of caregivers with no access to internet. Thus, the experiences of those who may have faced greater challenges were not documented. Lastly, the data collected is based on self-report and may be subject to recall and social desirability bias.

Future research should: (1) explore caregivers’ reasons for not receiving services and help-seeking trends to inform interventions designed to facilitate service access and use, (2) explore the relationship between service knowledge and service receipt, and (3) examine sociodemographic disparities in receipt of services by status in Canada (e.g., refugee etc.) given caregivers in our sample were primarily Canadian and underprivileged groups often fare worse. In addition, a longitudinal follow-up study to describe service receipt and needs over the course of the pandemic would be informative.

## Conclusion

Our findings point to the subgroup of children that is at risk of not receiving clinical health care services during public health emergencies and to the inequitable impact of the pandemic on this subgroup of children. Addressing inequity in receipt of services means that health decision-makers respond to the needs of this subgroup through targeted actions and initiatives to reduce disparities. Children with developmental disabilities and delays have a right to enjoy the highest attainable standard of health and to be protected in emergency situations [67]. Only through a human rights approach to emergency planning that includes engaging vulnerable populations (e.g., those with disability) in the process [69], will emergency responses be accessible and inclusive.

## Data Availability

The data are not publicly available due to privacy and/or ethical restrictions (participants did not provide informed consent for broad sharing of the data collected). However, data are available from the data access committee at Elsabbagh Lab (autism@mcgill.ca) for researchers who meet the criteria for access to confidential data.

## List of abbreviations

COVID-19: Coronavirus disease of 2019
DDD: Developmental disabilities and delays
Sars-Cov2: severe acute respiratory syndrome coronavirus 2
WHO: World Health Organization
UNICEF: United Nations Children’s Fund
OR: Odds ratio
CERB: Canada Emergency Response Benefit
CRCB: Canada Recovery Caregiving Benefit
UN: United Nations

## Declarations

The Research Ethics Office (Institutional Review Board) of the Faculty of Medicine and Health Sciences at McGill University approved this study and all participants provided informed consent to participate. The authors confirm that all study procedures were performed in accordance with relevant guidelines and regulations including the Declaration of Helsinki.
